# Medico-Chirurgical Transactions, Vol. XII. Part II

**Published:** 1824-03-01

**Authors:** 


*Medico-Chirurgical Transactions.* Vol. XII. Part II. 8vo, pp. 589, with coloured plates, price 18s. in boards. London, October 1823.

When the Society promised a part of a volume every half year, we doubted the propriety of the promise, and the power of fulfilment on the part of the Society. It is now more than a year since the first part of this volume was published, and we commend the committee of publication for waiting till their materials were ample, rather than coming forward with a light cargo. Although the present part is inferior to many, it is equal to some of its predecessors. It has the merit also of presenting a great variety in the bill of fare —no less than forty-three articles. It is a great pity, we think, that the quality of the plates should so enhance the price of the work as to render it beyond the reach of thousands of the lower orders of the profession. The medical journals, however, remedy this evil to a considerable extent; and, in order to aid in this useful operation, we shall give a very ample analysis of the articles in the order of their standing.

Article I. *An Account of two Cases of Biliary Calculi of extraordinary Dimensions.* By T. Brayne, Esq.

*Case* 1. A widow woman, 55 years of age, of spare habit and melancholy temperament, had enjoyed uninterrupted good health for many years prior to the last twelve months, during which period she had felt such uncommon languor and prostration of strength as to be rendered unfit for her occupation, that of nursing the sick. About six months before the date of report she was seized with paroxysms of pain in the epigastrium, generally commencing towards night, and continuing several hours. These returned every two or three weeks, for five or six times successively, and generally succeeded by slight jaundice, which commonly disappeared in a short time, leaving the patient in her usual health. About a month after the last attack she became the subject of common fever; and on the 26th of October, 1820, our author found her with small quick pulse, foul tongue, insomnium, constipated bowels, scanty high-coloured urine, and great mental irritability, amounting almost to insanity. She complained of no local pain; and in this state she continued, with little variation, till the 29th of November,* when she was suddenly seized with severe pain in the left iliac region, accompanied by tenderness on pressure. This urgent symptom continued unmitigated for sixteen or eighteen hours, ending in sudden ease, and presently afterwards a natural alvine evacuation, containing a calculus of extraordinary size. The febrile symptoms soon subsided, but the patient remained in a state of low muttering melancholy for some months, from which she at length recovered under the gentle and long-continued influence of mercury. The calculus resembled a pigeon's egg in form, but was somewhat larger. The surface was rough and tuberculated, some of the largest elevations being streaked longitudinally. When broken it exhibited a radiated and shining fracture throughout the facettes of the radii, but without any concentric laminated arrangement. For the details of the chemical composition of this calculus we must refer the curious reader to the original volume. There can be little doubt of its being of biliary origin; but the question is—did it pass the biliary ducts in the shape and of the size it exhibited when expelled the body? Although the thing is not impossible, it is very improbable. In this case, the subsequent death of the patient proved that ulceration was

* The statement is from the 26th of November till the 29th of October, which is manifestly an error.

the process employed by Nature to open a passage for the extraneous body. An adhesion was discovered between the gall-bladder and the duodenum, the size of a shilling, and in the centre of this adhesion was an aperture that would admit a crow quill.

*Case* 2. Grace Adams, 65 years of age, demanded Mr. Brayne's instant attention, on the evening of the 24th of February, 1822, when he was informed by her friends that she had been entirely obstructed in her bowels since the 19th, although various means had been tried by her medical attendants. She was lying on her back, her abdomen prodigiously inflated, the extremities cold, the pulse scarcely perceptible. Although her articulation was indistinct, she was yet sensible, and her countenance did not exhibit the true Hippocratic features. She vomited incessantly, and had rejected every thing from her stomach since the 19th, when the sickness had first come on, without apparent cause. Three drops of croton oil, in two pills, were ordered, one to be taken immediately, and the other in four hours, if necessary. The second dose was taken; but on the following morning the bowels had not been relieved, though the stomach was more quiet than it had been. Castor oil—colocynth and small doses of opium. In the evening some thin, offensive, dark-coloured stools had been procured, and the more threatening symptoms were subsiding. Various purgatives and injections were persisted in, and, after two or three days, evacuations more natural in colour and smell were freely passed. The figured motions were small and somewhat flattened, which induced the apprehension of a stricture, but in a day or two they became of their natural size. From this time the patient rapidly improved, and soon left her bed. A few days after this period she suddenly passed by stool, after some tenesmus and straining, a calculus; and in the course of the next six days another, both of the biliary character. The first calculus voided is of a singular form, being somewhat of a flat square shape, with its angles rounded, and its two sides hollowed out as if compressed when soft by some convex body. Our author considers the composition of these calculi to be of the same nature as that of the majority of biliary concretions.

"A comparison of these cases will lead to several interesting points of remark. In some respects they run nearly parallel. The sex and ages of the patients;—the comparatively slight degree of hepatic irritation excited;—the nocturnal returns of the pain in the epigastrium; the trivial jaundice in one, and the total absence of it in the other;—the severity of the symptoms of the intestinal obstruction;

—and the subsequent dropsical affection of the thorax, are obvious coincidences. The calculi in some respects are very dissimilar, though they have many properties in common. In that obtained from the patient Woodward, the cholesterine appears nearly pure, and its radiated arrangement is not more curious and beautiful, than difficult to conceive in its origin. The deposition by laminæ may very naturally be expected to result from some peculiar progressive effects of a vitiated and stagnant secretion. But on the formation of the radiated example I am incapable of proposing an explanation." 267.

That biliary concretions may be formed in the duodenum, into which the bile is immediately poured, we have strong reason to believe, not only from the size of some calculi passed, but from the circumstance of their being often unattended by jaundice, which could hardly be the case, had they proceeded along the biliary ducts. That they occasionally find a direct ulcerated road from the gall-bladder into the gut, there can also be no doubt, from the appearances observed on dissection.

Article II. *On the Influence of Local Irritation in the Production of Diseases resembling Cancer and other Morbid Alterations of Structure.* By Henry Earle, Esq. F.R.S.

The surgeon, as Mr. Earle observes, is often placed in trying situations, when called upon to decide on the propriety of putting his patient to a painful and hazardous operation, or abandoning him to his fate, without hope of remedy.

"This anxious and most painful alternative applies not only to cases of extensive mechanical injury, where we are often called upon, without any previous knowlege of the powers of the constitution, to decide between mutilation and probable destruction, but likewise to cases, which from their progress and character, bear a close resemblance to those malignant affections, which experience teaches us are too deeply rooted in the constitution to be eradicated by any local operation. It is to the latter description of cases that I wish to call the attention of the Society," 269.

Our author remarks that the almost certain recurrence or co-existence of disease in remote parts of the body, and particularly in some vital organ, after the removal by operation of fungoid affections, has induced many practitioners to avoid operating altogether under such circumstances. Such decision Mr. Earle highly approves. The same observations, he thinks, will hold good in most cases of carcinoma, where the constitution is evidently tainted—although in these latter cases it may sometimes be expedient to obtain temporary ease and prolongation of life by the infliction of a tractable wound. Not unfrequently, on the other hand, says Mr. E. we meet with instances of disease, at first sight, so formidable as to deter from any operation, but, on enquiry, found to have originated in some local irritation, by which the morbid action is still maintained. In most of these cases, the constitutional disturbance is produced by the local pain and excitement. Our author has met with several instances of this description, in which patients have been rescued from the gradual inroads of a lingering disease, and perfectly restored by extirpation of the local malady. Some of these cases he proposes to adduce, and at the same time to point out certain circumstances which, he thinks, have not met with the attention they merit.

### On Diseases of the Lips.

Any morbid process, Mr. Earle remarks, in the neighbourhood of the external apertures of the body concerned in the operations of ingestion or excretion, is liable to assume an indurated and unhealthy aspect from the continual irritation to which it is subjected in the diurnal performance of the functions of the part. Thus, ulcerations about the mouth are often extremely difficult to cure, and, if neglected, will assume the indurated character of carcinoma. In advanced life, they will frequently exhibit a very unpromising aspect, and induce the surgeon to form a most unfavourable prognosis. Mr. E. has seen several of these, and the event in all but one, has led him to believe that they are not carcinomatous, or dependant on any constitutional disease, but on local irritation. Under the rigid discipline hereafter to be mentioned he thinks they may, if taken early, be almost always cured; but commonly, they do not fall under the surgeon's eye, till the disease has made such progress, as renders it most safe to remove it by operation, as in the following instance.

*Case.* Mr. Webb, aged 64, consulted our author in June 1816, for a disease at the angle of the mouth, which began in December, 1814, with an induration which soon ulcerated, and had since continued to make progress unchecked by any application.

"At the time of his calling upon me, the right angle of the mouth had ulcerated so far as to allow the lip to fall and to admit of a constant flow of saliva. The ulcer had a very irritable appearance, and occasionally bled, and around this, to a considerable extent, the cheek had a very firm scirrhous feel, and the glands under the jaw were enlarged. He suffered constant severe pain, and was scarcely able to introduce any food into his mouth, from the swollen state of the lips, and the pain it caused him. The discharge had a most fetid smell, and affected his breath so much as to make it very unpleasant to be near him. On minutely examining the part, it appeared to me possible to remove the whole of the disease, and by taking away the teeth in the lower jaw, which were rendered useless by the loss of all the corresponding ones in the upper, that the cheek and remaining portion of the lips might be brought into contact. I represented to him that I could not answer for the success of this operation, that every thing would depend on being able to obtain union by the first intention, and that if we failed in this, it was more than probable the wound would never heal: I further stated that if we succeeded in closing the wound, the relief might only be temporary." 273.

Under these disadvantageous circumstances, Mr. Earle removed the whole of the disease by an operation, after which the patient was not permitted to speak a word, eat, or drink, except by an elastic tube. Nothing sinister occurred during the treatment—the whole healed by the first intention, and in ten days the patient was sufficiently restored to leave his house. He has since continued to enjoy good health. In four cases of less extensive disease of the lower lip, having all the same threatening aspect, Mr. E. has removed the parts with perfect success. It has been remarked indeed, by all surgeons, that carcinomatous affections of the lip are removed with far greater prospect of success, than similar affections in other parts of the body.

"From my own experience, and from witnessing the success of others, I may venture to affirm, that few cases afford greater promise of success than those corroding ulcers, with scirrhous edges, which occur about the lip. The operation is so simple, and the wound so constantly unites by the first intention, that in cases that do not readily yield to local and constitutional treatment, it is far better to resort to it, and often the deformity will be less than when the ulceration heals without any operation. One circumstance may be worth adverting to, both in operating for the removal of a diseased portion of lip, and in the hare-lip operation. It is better to introduce first, the pin that is nearest the edge of the lip, the exact coaptation of the red margin of the lip is thus rendered more certain, and where this is nicely attended to, it is hardly possible, at a slight distance, to perceive that any operation has been performed." 277.

Cases occasionally occur, under peculiar states of constitution, when the application of stimuli, which have been previously harmless, will produce diseases of a very anomalous nature, as the following case communicated by Mr. Stanley will shew.

A young gentleman, aged 19, met with a severe accident, attended with a considerable loss of blood, which reduced him to a very weak state. Being induced during convalescence to smoke a segar, their arose, in two days afterwards an incrustation about the size of a sixpence, formed upon each lip at the parts which had been in contact with the segar. These incrustations were soon detached, and two sores were discovered beneath them, circular and excoriated, with matter on their surfaces. The absorbent glands under each side of the jaw swelled, and seemed disposed to suppurate, with disturbance of the general health. In five weeks after their appearance, they spontaneously healed; after which, eruptions of a papular character, appeared over the whole face and neck. These gave way under the use of alterative doses of the blue pill and sarsaparilla, but the disturbance of the general health did not subside for a considerable time.

"The case," says Mr. Stanley, "seems to present an instance of a vegetable poison producing sores that would, under any other circumstances, have been regarded as syphilitic, or, at least, as belonging to the pseudosyphilitic form of disease; these sores, irritating the absorbent glands in their neighbourhood, and affecting the system; the latter conclusion seems justified by the appearance of eruptions after the healing of the sores." 279.

*Integuments of the Nose and Face.* Affections resembling cancer are not very uncommon in these parts, and our author has successfully removed them by operation. Their causes may sometimes be traced to the irritation of snuff, shaving, but especially the vicissitudes of the weather.

"In all these diseases occurring in the integuments of the face in persons advanced in years, if the character does not improve under proper treatment, it is adviseable to remove the disease, which may be done in a large majority of cases with every prospect of permanent success." 282.

*Tongue.* Diseases of this organ assuming the character of carcinoma are generally submitted to the knife; but, in our author's mind, doubts arise as to their probable nature. "The period of life at which many of them have occurred, and the frequent success which followed the operations, are alone sufficient to create a reasonable scepticism of their being cases of carcinoma." Our author does not call in question the propriety of such operations, where the whole of the morbid part can be removed with safety. Such operations, however, are far more formidable than when the life is affected.

"Not unfrequently, however, cases occur in which the disease includes parts which are beyond the reach of any operation. Under such circumstances, it is most fortunate that many of these affections will be found to yield to a proper combination of local, with constitutional treatment; even where the characters of the disease bear a very close analogy to carcinoma. Under the head of local treatment, I would place in the first rank, the removal, as much as possible, of all local stimuli, such as the taking away any decayed or projecting tooth, the shielding the tongue from pressure by covering the teeth with wax or soft lint, the *complete privation of the faculty of speech*, the frequent cleansing of the mouth with a stream of water, or any medicated liquor from an elastic gum bottle, instead of gargling or washing the mouth by any muscular effort, the employment of the most mild unirritating food, and, in bad cases, the prohibition from the use of solid food, substituting, in its room, milk and strong broths, which may be thrown into the stomach through a tube passed down the œsophagus, and even, in some cases, introduced through the nostril. Such a plan of treatment requires much firmness and resolution on the part of the patient: but I can confidently state, that it will be occasionally followed with success. When there is any enlargement of the glands, it will be right to combine with this the repeated application of leeches under the chin. As local applications to the ulcerated surface, few are more efficacious than a solution of nitrate of silver, or very much diluted nitric acid, in the proportion of three or four drops to the ounce. Occasionally a solution of arsenic is very useful. Any of these may be thrown upon the ulcer with a syringe, which will be found better practice than keeping lint moistened with any lotion, constantly applied to the part."* 285.

*Prepuce.* It is generally in old people that the deleterious influence of the constant application of local stimulants about the prepuce appears. When the disease is far advanced, it sometimes includes the glans penis, or becomes so connected with it round the corona glandis, as to render it necessary to remove that body, together with the diseased integument.

"The following appears to be the history and progress of this affection, which has been generally considered as cancer of the penis. It invariably occurs, as far as my observation goes, in persons with elongated foreskins. A want of proper attention to cleanliness in

---

* "This plan of cleansing wounds with a stream of water thrown on the part from an elastic gum syringe, I have very frequently found extremely useful, not only in ulcers of the tongue, but in any painful ulcerations about the tonsils or fauces, in which cases it frequently happens that the effort of gargling is productive of great pain, and is very insufficient to remove the sloughs, and inspissated sputum, which is often very tenacious and difficult to be got rid of. All that is required, is for the patient to hold his head over a basin, with his mouth open, and by compressing the elastic bottle, he will throw a stream with considerable force on any part of the throat to which he may wish to direct it. This may be readily done by patients in the most enfeebled state."

removing the secretion from behind the corona glandis, will often lay the foundation of this complaint. The prepuce, from the confinement of this irritating matter, is excoriated, and what at an earlier period of life would be called gonorrhœa præputii is the consequence, the part becomes œdematous, the frænum thickened, and it is soon impracticable to withdraw the foreskin. Phymosis being thus established, the frequent passage of the urine over the inflamed skin, causes it to ulcerate, and the continual application of so stimulating a fluid, produces much surrounding swelling and induration. Not unfrequently, the natural opening in the prepuce becomes nearly obliterated, and the urine dribbles away through several ulcerated apertures. An intractable disease is thus established, for which, when it has attained this height, the knife is the only remedy." 288.

The operation may be resorted to even when the characters of the complaint have assumed a very malignant aspect, and with confident expectation of success.

"If taken at an earlier period, the disease is perfectly remediable by removing the exciting causes. The greatest attention to cleanliness is the first requisite, and for this purpose, an elastic gum syringe, with a blunted pipe, should be frequently employed to wash away the secretion from behind the corona glandis, and to convey any medicated lotions to correct the morbid action which has been excited. Where phymosis is quite established, and the opening is much contracted, an elastic gum catheter should be introduced, and if no disease in the urethra or prostate gland prohibit the practice, it should be retained in the bladder. The irritation from the constant flow of the urine, will be thus effectually prevented; and, generally speaking, the swelling and hardness will subside sufficiently to enable the patient to withdraw the skin to admit of the exit of the urine without passing over the prepuce. The introduction of a small portion of sponge will often assist much in dilating the contracted orifice; but where these means do not succeed, it will be right, in some cases, to divide the prepuce, and expose the glans penis. I have known this operation succeed perfectly in a case which had been condemned to amputation. No operation should, however, be attempted, until the soothing plan of treatment has been tried, and the part brought into as quiet a state as possible; nor until the disease in the urethra, if any exist, be remedied as far as may be." 290.

Mr. Earle thinks, that in many cases where the whole of the penis is removed, the good effects would have been secured by a removal of diseased integument only. The contraction of the urethra, after operations of this kind, is often very troublesome, and, he imagines, might probably be obviated by a small slit being made in the urethra after the circular incision.

The soothing treatment, Mr. Earle observes, which has been recommended in these local diseases, may appear, and may sometimes really be, inadequate to counteract the morbid action going on. In many cases, however, he feels convinced it will succeed. No possible harm can result from a trial being made, as, in cases which may ultimately require to be extirpated, it is most desirable to allay irritation by every possible means, and none is so powerful as a state approaching to uninterrupted rest, as is every day witnessed in the beneficial influence of rest in diseases of the joints and vertebræ.

*Chimney Sweepers' Cancer.* Mr. Pott has left us a concise and accurate history of this curious disease. It is, according to Mr. Earle's opinion, invariably produced by the irritation of soot applied to the rugæ of the skin.

" The characters of the complaint are peculiar, commencing in a warty excrescence, which, in some cases, will remain nearly stationary for several months, or even years. After a time, the excrescence discharges a thin acrimonious ichor, which excoriates the surrounding skin. Ulceration now takes place in the centre, and the edges of the wound become everted, and throw out a luxuriant growth, with scirrhous hardness, which discharges a very fetid irritating matter. The most common situation for this complaint is the lower part of the scrotum; but this rule is not without exceptions: a remarkable instance of its occurrence at the wrist of a gardener, who was every spring employed to distribute soot for the destruction of slugs, is related by my late father in the last edition of Pott's works. Some instances have also occurred where the disease has taken place in the integuments of the face. As the disease advances, the parts immediately contiguous become affected, it spreads downwards to the perineum, and, if not arrested, will include the whole scrotum; at the same time, it not unfrequently extends itself to one or both testicles. When the testicle is first affected, it becomes firmly connected with the diseased scrotum; it enlarges, and becomes greatly indurated; ulceration, and sometimes sloughing, then take place, leaving a deep excavated ulcer, that penetrates into the body of the testis, which does not appear disposed to the formation of fungous growth, similar to what occurs when the scrotum is the seat of disease. The same observation applies when the complaint has extended itself to the inguinal glands; its progress in glandular structures appears to be more rapidly destructive without the slightest effort at reparation The disease, in every instance that I have seen, except one, extended itself to the parts immediately contiguous. The inguinal glands are often enlarged, but they will generally subside on the removal of the diseased scrotum; clearly proving that the disease is not commonly communicated in the course of the absorbents. This is a very important feature in the complaint, and one which most materially influences the prognosis and treatment. I know but one exception to this rule, where a bubo formed, which suppurated, and the sore assumed the same characters as the primary affection in the scrotum. Even where the testicle is affected, the disease is confined, for a time, to that gland, but it subsequently extends itself up the spermatic chord, into the cavity of the abdomen, where it proves rapidly destructive. Not only is the discharge from the sore very fetid, but the perspiration from the whole body, which is generally copious, has a very peculiar ammoniacal odour, which cannot be mistaken when once recognized. The countenance has a peculiar leaden hue, and the general health is materially affected by the severity of the pain, the want of rest, and the constitution having to contend with a disease which it is incapable of throwing off." 299.

This disease rarely attacks under the age of 30 years—which accounts for its comparative infrequency. In addition to this, our author is disposed to think that "a constitutional predisposition is required, which renders the individual susceptible of the action of the soot." According to Mr. Earle's experience, no topical applications or internal remedies have the slightest influence over the disease. The scalpel is the only resource, "and that may be resorted to with the most confident expectations of success, provided the whole of the disease can be removed." Even when the inguinal glands are enlarged, this, he thinks, ought to be attempted. "Where the testicle has become affected, provided the spermatic chord has not participated, it will be right to give the patient the chance of recovery rather than abandon him to a miserable and painful death." In two instances of this kind, Mr. Earle has known the operation to succeed, contrary to the opinion of Pott.

Our author has appended two cases to this paper, but we need not stop to analyse them here.

ARTICLE II. *On the Destruction of the Fœtal Brain.* By Mr. HAMMOND.

This case is as curious to the physiologist as it was distressing to the parties concerned at the time of its occurrence. A young woman, having an unusually narrow pelvis, could not expel the fœtus by the usual means, and it became necessary to open the head. The skull was perforated near the fontanelle, and a portion of parietal bone was removed. Three fingers were introduced into the brain of the fœtus, the cerebrum was "completely broken down, and about two ounces of brain taken out. The head and the whole of the fœtus were then quickly extracted."

"The child immediately cried heartily, and breathed in a perfectly healthy manner, and with the usual apparent strength. It bled considerably from the wound in the head; a dossil of lint was applied, and kept on by adhesive plaister. This seemed to moderate, but not to stop the hæmorrhage; the child passed fæces and urine, and for twelve hours the functions of life seemed to be carried on in the usual and healthy manner; the child frequently crying loudly. After that time it became weaker, cried more feebly, looked very pale, and seemed gradually sinking; for ten hours it continued in this state, during the last two it was slightly convulsed. The bleeding from the wound, though not violent, never ceased, and the child appeared to die more immediately from the hæmorrhage.

On examining the brain after death, I found the dura mater considerably torn, the cerebrum throughout both hemispheres torn and broken down, so as to appear a mere mass withont organization; where the ventricles should have been was filled by clotted blood. The cerebellum, medulla oblongata, and spinal marrow were not injured. The child lived forty-six hours." 309.

This was a most distressing scene for the mother; and the practitioner must have often been reminded of Macbeth's exclamation—

"—————— Time was,
That when the brains were out the man would die."

The case shews that the removal of a part, and the breaking down of the whole cerebrum do not ensure immediate death to the fœtus. In such cases, our author thinks it would be better to divide the medulla oblongata or the spinal chord. We imagine it would not be very easy to divide the spinal chord, at the time we are opening the head. The medulla oblongata might be destroyed by the perforator, or even the finger.

Article III. *A Case of Bronchocele.* By Henry Shuckburgh Roots, M.D. Physician to the Carey Street Public Dispensary, &c.

This case is brought forward with the view of illustrating the advantage to be obtained from the use of iodine in bronchocele.

*Case.* A young lady, 19 years of age, of scrofulous disposition, had had, for two years, an enlargement of the thyroid gland, and which had increased rapidly within the last four months, occasioning a good deal of uneasiness, especially when bending the chin, singing, or laughing. The tumour was equal in size to a flattened orange. Eight leeches were applied twice a week, and 15 grains of the carbonate of soda thrice a day. This plan was persisted in for three months, without any material alteration in the size of the gland. She was now directed to take a drachm of the burnt sponge*three times a day, and to apply the leeches once a fortnight. This plan was also adhered to for about three months, but still no reduction of the tumour was effected. Frictions, with liniments, and a continuance of the burnt sponge, were next employed, but without success. The iodine ointment, nearly in the form used by Dr. Coindet, was then applied, the size of a garden bean being rubbed into the tumour night and morning.* She continued the use of this ointment for five weeks, during which time no pain was experienced in the tumour, and some dimunition of the size of it was produced. The ointment was now increased in strength, from 34 to 44 grains. Inflammation took place in the integuments of the tumour, and leeches were ordered in lieu of the ointment. The inflammation being subdued, she resumed the use of the ointment, and continued it till the latter end of July, when the tumour on the left side was entirely dispersed, and that on the right much diminished. The tincture of iodine was now exhibited (20 drops thrice a day) in addition to the ointment. She took the iodine twice a day internally for six weeks, and then once a day till near December 1822, when the tumour in every part was entirely dispersed.

We beg again to caution our brethren against the internal use of iodine. It has been productive of fatal effects. The external application is a powerful mean of reducing glandular tumours of all kinds.

Article IV. *On the Dilatation of the Male Urethra by Inflation, for extraction of Calculi from the Bladder, as practised in Egypt, near* 250 *Years ago.* By Robert Masters Kerrison, M.D.

When, in the 14th Number of this Journal, we exposed the absurdity of Prosper Alpinus's account of the extraction of calculi by inflation, we had no idea that this account was publishing in the Transactions of the Medico-Chirurgical Society. We had heard, indeed, that the passage had been quoted by some person, and we appealed to the Society afterwards, *viva voce*, whether the whole story did not involve an anatomical impossibility, and was not, consequently, a *juggle?*

* In a late conversation which we had with Dr. Coindet at Geneva, he informed us that he now never administers the iodine internally, as it is productive of a strange and peculiar disease of the system, apparently from its too great action on the absorbent vessels. The ointment he uses is composed of hydriodate of potass half a drachm, hog's lard one ounce and a half, white wax three drachms, liquor potassæ caust. gt. ij. It is to be rubbed twice a day on the part affected, for eight days, then stop for eight days, and begin again, and so on. Leeches, he says, greatly promote the effects of the ointment.—*Rev.*

Not one in the Society attempted to defend it—even the Medical Intelligencer has been candid enough to avow, that when he transcribed the passage from Prosper Alpinus, he "*never meant to convey that the operation, as described by Alpinus, could have been performed*." "It would, (says he) have been in the highest degree *absurd for us to have thought so*."* Why then, in the name of common sense, bring up what are acknowledged to be fictions or impossibilities, as anticipations of modern inventions? We are really surprized that Dr. Kerrison, of all others, should have been so deceived, or should have considered the relation of Alpinus with so little reference to anatomy, as to single out this story "as a point of early surgical *practice*, now submitted for the consideration of the profession." We repeat it, the whole is an anatomical impossibility.†

As for the operation of Hildanus, it is for the extraction of calculi *lodged* in the urethra, and the forceps, of which he has given a drawing, are like a pair of slender sugar-tongs, bearing no resemblance, in structure or use, to those invented and employed by Sir Astley Cooper and Mr. Weiss. In order to make the current of our analysis more uniform, we shall here bring forward an article which, though read at the Society long *before* Dr. Kerrison's, is yet placed several articles *later* in the volume of Transctions. The reason of this we do not pretend to divine.

Article V. *Further Account of the Extraction of Calculi from the Bladder, without the Use of any cutting Instrument*. By Sir Astley Cooper, Bart.

We have been a good deal astonished at the running title which has been placed over this article in the Transactions; namely, "on the extraction of calculi by *dilatation* of the urethra." There is nothing in the title of the MS. paper to authorize this heading; and, transposed as the article now

* Med. Intelligencer, for September, 1823, p 505.

† Not having been present at the Society the night Dr. Kerrison's paper was read, we knew not of its existence, and, consequently, no remark which we have made on the operation of Prosper Alpinus, has any reference to that gentleman, whom we have long respected, though personally a stranger to us, as a very enlightened, learned, and amiable member of medical society. We had heard, as before observed, that a *viva voce* reference had been made to Alpinus, and we made a *viva voce* reply. Afterwards, we saw the passage quoted in the Medical Intelligencer, and then we made a written remark on it. In all this, we acted with that candour and independence which, we trust, we shall maintain as long as we have the honour of addressing an enlightened public.—*Rev.*

stands, with the forced title over its head, it appears to the reader as an appendix to the romance of Haly the Arab! yet it is not a romance, as we shall soon shew.

In a former volume of these Transactions, Sir Astley hinted an idea which occurred to him, that small calculi might be extracted from the bladder by forceps introduced through the urethra. Such forceps is now well known, and reflects much credit on the ingenuity of Mr. Weiss, its constructor, and, partly, its inventor. From the Reverend Mr. Buller, Sir Astley extracted more than eighty calculi, and, since that time, our author and others have extracted more.

*Case* 1. Mr. Brodie has communicated to Sir Astley Cooper the particulars of a case of this kind. A gentleman, aged 70 years, had frequent desire to make water, with more or less difficulty in voiding it, sometimes requiring the use of the catheter. On introducing the sound, Mr. Brodie distinctly felt some calculi, previously to the sound's reaching the bladder; and, on examination being made by the rectum, a number of calculi were felt in the situation of the prostate gland, apparently contained in a cyst, and sliding on each other under the pressure of the finger. On the first introduction of the new forceps, Mr. B. removed two calculi only; but, in the second attempt, six or seven were brought away of a larger size. The operation was repeated ten or twelve times, at various intervals, in the course of six weeks, and, in all, about 60 calculi were extracted. The largest measured half an inch in one diameter, and five eighths of an inch in the other, having four sides and angles. At the end of July, the symptoms were very much relieved, and no more calculi could be discovered:—But, having undertaken a journey, he was again seized with difficulty in voiding his urine, and, on returning to town, another calculus was extracted from the membranous part of the urethra by the same instrument.

*Case* 2. Sir W. B. aged 67, had suffered from long and repeated attacks of gout, from the 35th to the 60th year of his age, since which, the attacks are less frequent, and much mitigated. It is seven years since he first passed gravelly concretions, and these have continued since that period. In 1820, after considerable walking exercise, he passed a quantity of what appeared to have been blood—and fast riding, at any time, induces the same discharge. Sir A. Cooper and Sir Gilbert Blane now attended the patient, and, on being sounded, it was discovered that there was a stone. As it appeared to be a small one, Sir Astley proposed a trial with the new forceps. On the fourth attempt, a stone, weighing 17½

grains, was extracted, on the 18th of July. Some time after this, another calculus was discovered, but, being too large, a dilatation, in this instance, was made of the urethra by the introduction of bougies. A stone was then extracted, weighing 54 grains, on the 28th of August. Some pain in making water, swelling of the corpus spongiosum, and urethral discharge, followed this last operation; but they subsided under the application of poultices and fomentations.

When the size of the stone is considered, it need not excite surprize, that much difficulty was experienced in the extraction. It was in that part of the urethra near the glans, that the chief impediment was found, and the stone could easily (if it had been thought necessary) have been removed from thence by incision. Upon after-thought, Sir Astley is of opinion that, in removing a stone of equal magnitude, it would be better to make a small incision into the uretha, anterior to the scrotum, than employ force for its extraction through this narrower part of the channel.

In a communication from Sir Gilbert Blane to Dr. Cooke, on the above case, Sir Gilbert observes that—"had it not been for the new method so happily conceived, and so skilfully executed, by Sir Astley Cooper, the patient would have been subjected either to the sad sufferings of the stone, or to the pain and danger of lithotomy."

*Case 3.* Mr. King, aged 66, mariner, came under Sir Astley's care on the 30th of October, 1822. On sounding, it was found that he had a calculus in the bladder. Sir Astley passed the long urethral forceps into the bladder, and, in a few minutes, extracted four calculi. On the 1st of November, he extracted three calculi—on the 4th, five more—on the 7th, twelve calculi—on the 11th, two—and, on the 13th, three more. On careful examination, no more stones could be perceived.

"It is delightful to hear the expressions of gratitude which this patient pours forth for the relief which he has experienced from these operations, under which he has suffered but a slight degree of pain, and has never, for a moment, been confined from whatever exercise he was disposed to take." 393.

From Mr. Brodie's patient two other calculi have been since extracted by the same means; and Sir Astley has lately removed from a young person, of the name of Errington, a calculus of moderate size, and enabled two others to pass, by withdrawing the instrument in its dilated state; in consequence of which the stones passed, in the afternoon of the same day, in a copious discharge of urine.

The stories which were circulated respecting non-originality of this instrument and operation, and the raking up of Haly's juggling tricks in Egypt, together with an exaggeration of Hildanus's forceps, have drawn forth some sarcastic remarks from Sir Astley, at the close of this paper.

"I have heard that it has been stated that there was no novelty either in this idea or in the instrument. To this I have only to observe, that if the idea had previously occurred to any individual, he had so far buried it in his bosom, that I had never heard of it; and as to the instrument, I am quite sure that Mr. Weiss consulted no musty volume for its formation, for so soon as I mentioned my wish he should construct a pair of forceps by dividing a sound in its middle, and giving it a joint two inches from its end, he, without quitting me, observed, that he should make them to open in the mode which the plate in the former volume represents. Mr. Weiss has a strong and ingenious mind, and does not use petty artifices to obtain employment or character. But let us for a moment suppose, (what I do not believe,) that the idea had occurred to others, and the instrument had been made centuries ago, what are we to say of the apathy of those bright ornaments of their profession, Cheselden, Pott, Hunter, Cline, Home, Blizard, &c. who, if they had heard of such an instrument, had never employed it?" 394.

We have already (page 480 of this vol.) pointed out the absurdity of Haly's operation; and as to Hildanus, it will be seen, at page 756, chap. 27, that that surgeon never contemplated the extraction of calculi from the *bladder*, but merely from the *urethra*, when they happened to get jammed in that passage. After directing the anterior portion of the urethra to be cautiously dilated, he introduces a slightly curved forceps —"tunc indice dextro calculum leniter propellit." It is needless to say that this operation has not the remotest resemblance to that of Sir Astley Cooper*

Article VI. *Cursory Remarks on Small Pox, as it occurs subsequent to Vaccination.* By George Gregory, M.D. Physician to the Hospital for Small Pox and Vaccination at St. Pancras.

Dr. Gregory's opportunities for observation are now extensive, and his zeal in medical science is unquestionable: we therefore look with much interest to this report on the present important subject. That the failures of vaccination have been

* Speaking of originality, we may here remark, that Hey's saw, as it is called, is most correctly delineated in table 32, figure 9, of Scultetus's Armamentarium Chirurgicum, where it is seen removing a portion of fractured cranium.—*Rev.*

steadily and progressively on the increase for some years past, no unprejudiced spectator can deny. "The difficulty," as Dr. G. properly remarks, "cannot be met by reference to the fact, that small-pox, once gone through, does not always secure to the subject immunity from a second attack." Cases of small-pox after vaccination are, beyond all comparison, more frequent than cases of *secondary* small-pox. Within the walls of the small-pox hospital these *last* are rarely seen, whilst "the *former* constitute, at the present time, a considerable proportion of the admissions into that institution." Dr. G. has given a table of the total number of admissions into the small-pox hospital in ten different years; and from this table it appears that, in the year 1810, the proportion of cases of small-pox succeeding vaccination to the whole number of admissions, was as 1 to 30—while, in 1815, it was as 1 to 17—in 1819, as 1 to 6—in 1821, as 1 to 4—and during the year 1822, as 1 to $3\frac{1}{2}$. This is a frightful ratio of progression; and we fear that it cannot be accounted for on any other principle than the actual increase of failures in vaccination.

Above 100 cases of small-pox after vaccination have occurred at the small-pox hospital during the last three years, the greater number of which fell under Dr. Gregory's observation—consequently his opportunities for inspection of this form of disease have been sufficiently extensive to sanction his laying the results before the profession.

Notwithstanding the state of the case as sketched above, Dr. Gregory has no fear for the cause of vaccination in general, however strict the inquiry into its power of protection be made.

"A large proportion of mothers, in the present day, were themselves vaccinated, and, therefore, the popular prejudices may now be considered as in favour of vaccination rather than against it. So far from anticipating evil, I look forward to the public good being benefited by the free discussion of the subject. Many persons have been brooding in secret over the failures of vaccination, and appear to have a fear of expressing their sentiments concerning it, or of meeting the question, in any way, openly. To them the avowed investigation of the subject will, I am persuaded, prove satisfactory. But, besides this, it is only by candid discussion that we shall ever be able to determine that highly important point, how far the failures of vaccination are owing to causes under our control; and how far, therefore, there exists a reasonable probability of obviating them, either wholly or partially, so as to increase the security of the vaccinated." 327.

Our author's first inquiry is, in what manner and to what extent the effects of the variolous poison are modified by the influence, previously exerted, of the vaccine virus.

It is hardly necessary, in the first place, to remark that, in a very large proportion of cases, vaccination affords the same immunity as the genuine variolous disease; but what the *exact* proportion is, we are unable to ascertain, because "the failures of vaccination, are now far more numerous than they were ten years ago, and no certainty exists, that they have yet reached their maximum."

Where perfect security is not imparted by vaccination, there is at least a modification "of *certain* of the effects" of variolous contagion. These modifications we cannot give in better terms than Dr. Gregory himself has used.

"1. Vaccination does not appear to lessen the violence, or shorten the duration, of the first or eruptive stage of fever, which is generally as severe, and even sometimes severer and longer in its duration than that of the casual confluent small-pox.

"2. It does not appear in like manner to influence the *quantity* of eruption upon the skin, so much, at least, as has been generally imagined. It is true that, in many cases of small-pox, subsequent to vaccination, the eruption has been very scanty; but, in a large number also, I have seen it very copious, more particularly about the face, breast, and upper extremities, and occasionally fully equal, in point of *quantity*, to what is seen in the worst kinds of confluent or coherent natural small-pox.

"3. The great power of vaccination unquestionably consists in modifying the *progress of inflammation* in the variolous eruption; and here it cannot fail to attract observation, how strikingly opposed to each other, in this respect, are the influences of inoculation and of vaccination. Inoculation lessens the *quantity* of eruption, but does not alter, in the slightest degree, the progress of inflammation in that which *is* brought out. Vaccination, on the other hand, while it does not sensibly affect the *quantity* of eruption, always influences, more or less, the progress of inflammation, however copious the eruption may be. The same desirable result, the diminution of mortality, is obtained in either way. By checking the quantity of eruption, or the degree to which inflammation in it extends, the disease is prevented from bringing on those impediments to the functions of respiration and perspiration, which occasion secondary fever, and endanger life." 330.

We cannot but think that Dr. Gregory is a little deceived in concluding, that vaccination does not sensibly lessen the violence of the eruptive stage, nor the quantum of the eruption itself. In the great majority of those cases of *variola post vaccinam* which we have seen, the violence as well as the quantum of eruption were greatly mitigated. Has this difference in our experience arisen from Dr. Gregory's having opportunities of seeing more of the severe cases in hospital practice than those private practitioners who see cases as they occur, without selection?—We imagine that there is something in this.

Our author observes that in all, or nearly all cases of natural and inoculated small-pox, the eruption proceeds to ulceration, more or less superficial, and the ulcers heal by the common process of scabbing and cicatrization. In *variola post vaccinam*, on the other hand, "the cutaneous inflammation is checked at so early a period, that the fluid in the vesicles seldom reaches the state of pus—the cutis vera is never ulcerated, and consequently the healing process takes place, by the conversion of the vesicles into tubercles, and their subsequent desquamation." This, he observes, constitutes a well marked and important character of the post-vaccine small-pox. Our author also has remarked, that though vaccination modifies, in a large proportion of cases, the progress of inflammation in the skin and throat, it does not always do the same "when the variolous poison fixes itself on other parts, more particularly on the brain."*

The *degree* of modifying power in the vaccine disease varies to an almost incredible extent, in different individuals. Sometimes the post-vaccine affection is so slight as to be confounded with varicella, or even the trifling papular eruptions of warm weather—at others, it is as severe as the genuine small-pox. Between these extremes, every possible gradation has been noticed at the hospital. It is gratifying, however, to know that the instances of *complete failure* are very few in number. Of 57 cases of small-pox *post vaccinam* admitted into the hospital in 1822, 44 were discharged in perfect health within 14 days. Five proved fatal; but their histories did not afford satisfactory proof of their having gone through the vaccine process in a proper manner.†

Dr. Gregory is convinced, from observation, that a disposition to post-vaccine disease prevails in particular families. Thus, he has seen a father and two of his children come in—the former with pretty smart modified small-pox, after *inocu-*

---

* "I may be permitted, perhaps, to remind those who have not been in the habit of seeing small-pox lately, that the eruption on the skin and throat is only one of the effects of the poison. Another, at least equally important, both with reference to pathology and practice, is that which is exerted upon the brain and nervous system; the chief evidences of which are delirium, inflamed eyes, stupor or restlessness, and disposition to erysipelas and gangrene." 331.

† Even this proportion of fatality is so much greater than is met with in private life, that we think our former observation on the difference between public and private practice is thereby strengthened.—*Rev.*

*lation*—the two latter with very slight forms of the same after vaccination.

It is curious, that a great majority of the cases of small-pox, *post vaccinam*, which have occurred at the hospital, have been in persons between the ages of 15 and 21. The age of 19 is the average of the whole. Our author is induced to think that there is something in the habit of body peculiar to this age, which renders the system more than usually disposed to suffer from the variolous poison.

"In any investigation of the causes of small-pox subsequent to vaccination, it would be improper to overlook the remarkable connexion that subsists between the degree of perfection in the vaccine cicatrix, and the violence of the secondary disease. This important fact was forced upon my attention by the results of the last year's experience at the Small-pox Hospital. It is, indeed, in opposition to the opinion entertained by several authors of acknowledged reputation; but the extent of my opportunities enables me to speak with much confidence on this point. When the scar on the arm is perfect,—that is, distinct, circular, radiated, and cellulated; but, above all, when it is small, so that it may be covered by a pea;—the secondary affection (if from peculiarity of habit, or any other less ascertained cause, it does occur,) will be slight, and hardly deserve the name of a *disease*.

"On the other hand, whenever the seat is large, and bears the marks of having been formed by high local inflammation, and wants the other distinctive characters just enumerated, the chance of small-pox occurring in after-life will be greater, and, *cæteris paribus*, there will be a stronger likelihood of its proving severe." 337.

This principle, he observes, receives a striking confirmation from what takes place in *re-vaccination*. When the cicatrix is perfect, it is impossible, or nearly so, to re-produce the vaccine disease in any thing like its genuine form. "In proportion to the imperfection of the cicatrix, will be the degree of approximation of the second to the primary vaccination."

"These considerations tend to establish, *as a pathological principle*, that the occurrence of small-pox, subsequent to vaccination, is dependent upon the *intensity of the vaccine influence, as primarily exerted;* and they lead to the belief, that the appearance of the cicatrix may be taken as a *measure of that intensity*." 338.

The last point to which Dr. Gregory wishes to draw the attention of the public is the fact that, a very large proportion of those persons who have been admitted, during the last three years, with variola *post vaccinam*, had been vaccinated *in the country*. Notwithstanding the objection to any inference being drawn from this, which was urged at the society, our author is still of opinion that this is not a mere accidental circumstance. We know indeed that the domestic servants in London (who are most naturally to be expected as applicants for an hospital when ill) are, in a great measure, supplied from the country. Still the disproportion between town and country vaccination is so great, that our author finds himself under the necessity of seeking for an explanation that may have a wider range of influence than the above. The explanation, he thinks, may be found in the deteriorated qualities of vaccine lymph in the country. This may be the case when the lymph is procured from a distance. But the generality of practitioners in the provinces vaccinate from their own or their neighbour's patients, and are just as good judges of the lymph as their brethren of the metropolis. We shall conclude this paper, which is of very great interest in itself, with the following extract:—

"If the notions which I entertain of the influence of vaccination on the animal economy, and on the causes of its occasional failure, are correct, their general diffusion might assist, not only in upholding vaccination as an object of great national and individual importance, but in checking those unpleasant occurrences which are now making such alarming inroads on the confidence of the public in the vaccine protection. It would induce practitioners to revaccinate, with fresh and genuine lymph, those whose arms do not exhibit the perfect cicatrix;—it would lead those in the country to apply more frequently to large towns for a supply of recent lymph;—it would point out the propriety of their putting less confidence than heretofore in points and preserved lymph;—and it might impress upon all the *indispensable* necessity of a close attentioa to every part of that process, which, though of trifling import at the moment, is yet of incalculable consequence to individuals in every future period of their lives." 340.

[*The Transactions to be continued.*]

