# Corrigenda in This Number

**Published:** 1824-03-01

**Authors:** 


					rage oUs, tor tiydroaate, read hydnodate;

				

